# Efficacy and safety of pyrotinib-based regimens in patients with HER2-positive stage III/IV breast cancer: a real-world retrospective study in China

**DOI:** 10.7717/peerj.20524

**Published:** 2026-01-13

**Authors:** Limin Zhi, Lei Huang, Xiaohong Pang, Qiong Wu, Yu Lei

**Affiliations:** 1 People’s Hospital of Guangxi Zhuang Autonomous Region, Nanning, Guangxi, China; 2 The Affiliated Tumor Hospital of Guangxi Medical University, Nanning, China

**Keywords:** Pyrotinib, HER2-positive breast cancer, Stage III/IV, Efficacy, Safety, Real-world study

## Abstract

**Background:**

Data from multiple clinical trials have shown that pyrotinib has demonstrated significant efficacy and acceptable tolerability in patients with HER2-positive advanced breast cancer (BC). However, the short time to market in China limits our comprehensive understanding of the drug’s long-term efficacy and potential adverse events (AEs) from the drug. Therefore, this study analyzed the clinical efficacy and safety of pyrotinib-based regimens in a real-world database.

**Materials and Methods:**

This study retrospectively analyzed patients with HER2-positive stage III/IV BC who were treated with pyrotinib-based regimens from October 2018 to October 2022 at the Affiliated Tumor Hospital of Guangxi Medical University. Tumor assessments were based on RECIST 1.1, and AEs were assessed and graded according to NCI-CTCAE 5.0. Long-term efficacy was evaluated by calculating median progression-free survival (mPFS, defined as the time from treatment initiation until disease progression or death).

**Results:**

Of the 37 included patients, the objective response rate (ORR) was 62.2%, the disease control rate (DCR) was 94.6%, and the median progression-free survival length was 12.0 months (95% CI [5.8 ∼18.2] months). A subgroups comparison found that significant differences were observed in patients who had not used lapatinib (*P* = 0.016), had a number of metastatic sites ≤ 2 (*P* = 0.011), were intolerant to trastuzumab (*P* = 0.004), and were on first-line pyrotinib treatment (*P* = 0.036), with these patients having median progression-free survival lengths of 13.0 months, 15.9 months, 23.5 months, and 23.5 months, respectively. Pyrotinib was also effective in patients with advanced brain metastases after multiple lines of complex therapy, with these patients having a median progression-free survival length of 5.0 months. Diarrhea was the most common adverse event (97.3%), with no grade 4 AEs observed. This study was the first to compare the relationship between different degrees of diarrhea and mPFS, and no significant differences in mPFS were observed (*P* = 0.291). In addition, a rare positive fecal occult blood profile (5.4%) was observed.

**Conclusion:**

Pyrotinib-based regimens have shown satisfactory clinical efficacy in HER2-positive stage III/IV BC patients, and pyrotinib is well tolerated with manageable adverse events.

## Introduction

Breast cancer (BC) is the most common cancer diagnosed in women, worldwide (Zheng et al., 2023). The incidence of BC in China has risen sharply (Xiong, Duan & Zuo, 2024). HER2-positive BC has a poor prognosis, while HER2 mutations are usually related to recurrence of BC and to drug resistance. The emergence of multiple new types of anti-HER2 drugs have greatly increased the survival rate of HER2-positive BC patients (Banys-Paluchowski et al., 2023).

Pyrotinib is a new type of irreversible small molecular tyrosine kinase inhibitor (TKIs) that can effectively target EGFR and HER2 to inhibit the development and progression of tumor disease (Li et al., 2017). In a phase II study, the objective response rate (ORR) of the pyrotinib group was significantly higher (21.3%) than the ORR of the lapatinib group, and median progression-free survival (mPFS) was also prolonged (Ma et al., 2019). Because of these significant phase II research results, pyrotinib was formally approved in China in 2018 for the treatment of HER2-positive BC, replacing lapatinib as a second-line standard treatment drug (Blair, 2018). Three recent phase III clinical trials (PHENIX, PHOEBE, and PHEDRA) also showed excellent efficacy data for pyrotinib (Yan et al., 2020; Xu et al., 2021; Wu et al., 2022). With its exciting performance in the recent PHILA study, pyrotinib was rapidly approved in China as the recommended first-line treatment for HER2-positive advanced BC (Ma et al., 2023b).

Although a number of clinical research studies have shown significant efficacy of pyrotinib in HER2-positive advanced BC patients and that pyrotinib is well tolerated, most of these studies are pre-market clinical trials. The efficacy and safety of pyrotinib in the real world are still unknown. In addition, pyrotinib has only recently been approved in China, and the real-world data have not been fully disclosed. The long-term efficacy of pyrotinib is difficult to observe, and it is still unclear whether a high incidence of AEs affect the drug’s efficacy.

In the 2024 Chinese Breast Cancer Diagnosis and Treatment Guidelines (CACA & CMA, 2024), trastuzumab deruxtecan (T-DXd), a new drug approved in 2023, was recommended for patients with failed trastuzumab treatment. However, based on the medical facts and drug accessibility and economy in China, pyrotinib is still the first choice for second-line treatment of HER2-positive advanced/metastatic BC. The national medical insurance of China now includes the application of pyrotinib combined with a trastuzumab and docetaxel (PyHT) regimen for advanced first-line treatment, providing a more appropriate treatment option for patients with advanced BC and further consolidating the position of pyrotinib in the treatment of HER2-positive advanced BC. This study retrospectively investigated the efficacy and safety of pyrotinib-based regimens for the treatment of patients with HER2-positive stage III/IV BC in practical clinical applications and analyzed the efficacy of pyrotinib-based regimens in different subgroups.

## Patients and Methods

### Study population

**Figure 1:**
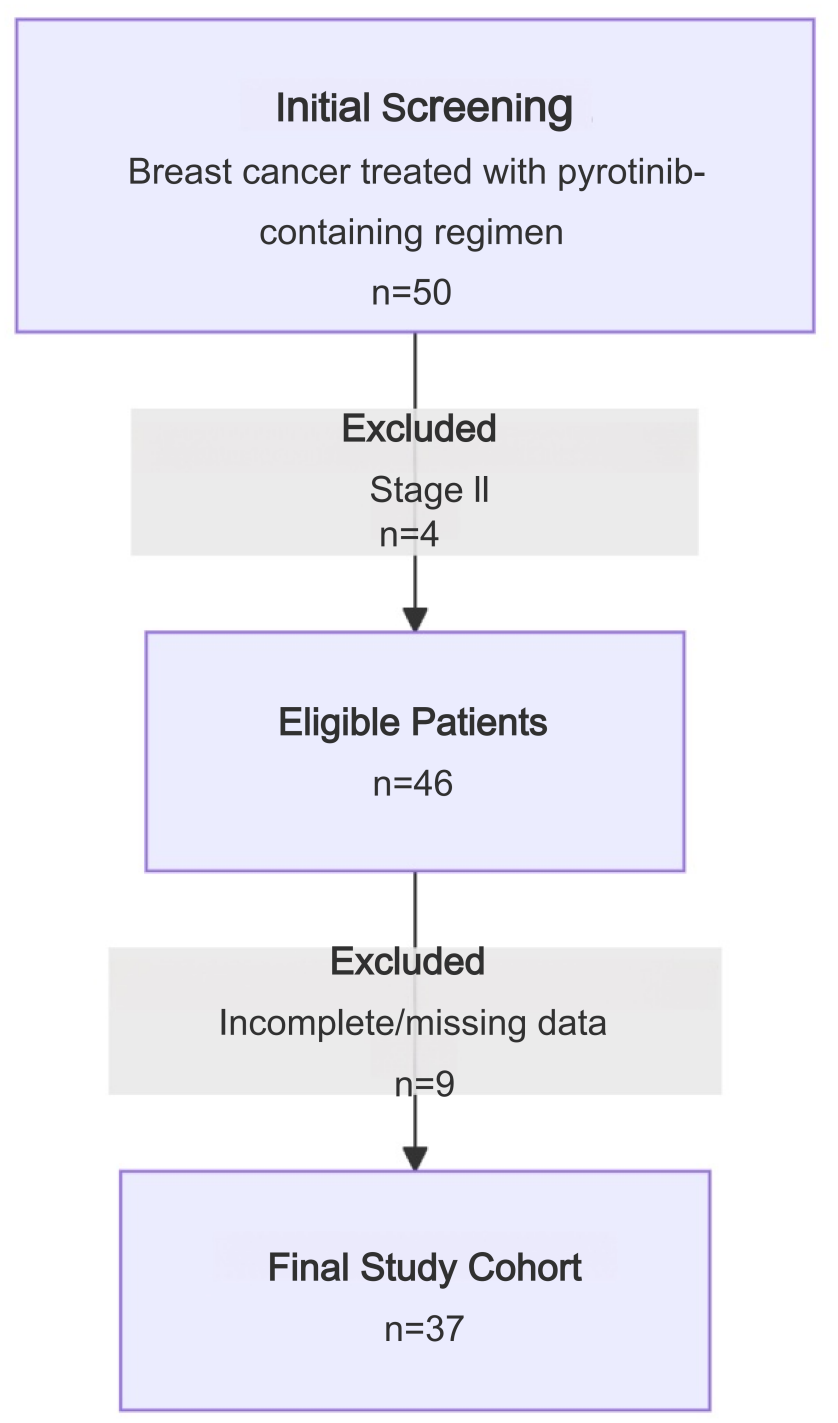
Study flow chart.

This study retrospectively collected medical records of patients with HER2-positive stage III/IV advanced BC treated with a pyrotinib regimen between October 2018–October 2022 at the Affiliated Tumor Hospital of Guangxi Medical University. Clinical data were collected from the hospital electronic medical record system. A study flow chart is shown in Fig. 1. Inclusion criteria: stage III/IV BC; the result of HER2 status was 3+ by immunohistochemistry (IHC) or 2+ and positive amplification by fluorescence *in situ* hybridization (FISH); and efficacy had been evaluated at least once after oral pyrotinib. Exclusion criteria: incomplete clinical data or loss to follow-up. A total of 50 breast cancer patients treated with pyrotinib were selected from the database. Four were excluded because they had stage II BC. Of the remaining 46 eligible patients, nine were excluded due to incomplete or missing data. Ultimately, 37 patients were included in this study (Fig. 1), with a median age of 50 years (age range: 29–79 years).

 This study adhered to the Declaration of Helsinki. The study protocol was approved by the Ethics Committee of the Affiliated Tumor Hospital of Guangxi Medical University, No. KY2023549. All participants were informed of the study and signed a consent form for participation.

### Treatments

Oral pyrotinib maleate tablets were to be taken by patients once a day in 21-day cycles until disease progression, unacceptable toxicity, or the doctor considered the drug to no longer be of benefit to the patients. Treatment regimens and dosages were determined by the physician based on clinical practice, patient physical condition, and patient preference. Radiologic evaluation (CT or MRI) was performed every 2–4 treatment cycles.

### Evaluation of efficacy and safety

The primary endpoint was progression-free survival (PFS), defined as the time from treatment initiation until disease progression or death. Secondary endpoints included ORR, DCR, and safety (measured based on the number and severity of AEs). ORR is defined as the proportion of eligible patients with complete response (CR) and partial response (PR), while DCR is defined as the proportion of eligible patients with CR, PR, or stable disease (SD). AEs were assessed based on NCI-CTC 5.0.

### Statistical analyses

SPSS 26.0 (IBM Corp., Armonk, NY, USA) was used for statistical analyses. Categorical variables were expressed as patient counts (and percentage of total). PFS was assessed using the Kaplan–Meier method and log-rank testing for between-group comparisons. All tests were two-sided (*α* = 0.05); *P* < 0.05 indicated statistical significance.

## Results

### Baseline characteristics

The baseline characteristics are shown in Table 1. Age: 18 patients (48.6%) were <50 years old, and 19 patients (51.4%) were ≥50 years old. Menopause status: 22 patients (59.5%) were menopausal, and 15 patients (40.5%) were pre-menopausal. ECOG (Eastern cooperative oncology group) performance: eight patients (21.6%) had an ECOG score of 0, 26 patients (70.3%) had an ECOG score of 1, and three patients (8.1%) had an ECOG score of 2. Hormone receptor (HR) status: 16 patients (43.2%) were positive, and 21 patients (56.8%) were negative. Ki67 values: 32 patients (86.5%) had Ki67 values ≥15%, and five patients (13.5%) had Ki67 values <15%. Clinical stage: two patients (5.4%) had stage III BC, and 35 patients (94.6%) had stage IV BC. Metastatic burden: 29 patients (78.4%) had ≤2 metastasis sites, eight patients (21.6%) had >2 metastasis sites, six patients (16.2%) had brain metastases (BMS), and 31 patients (83.8%) had visceral metastases. Previous anti-HER2 condition: 29 patients (78.4%) had received anti-HER2 therapy, including trastuzumab (*n* = 25), trastuzumab and patuzumab (*n* = 2), trastuzumab, patuzumab and lapatinib (*n* = 1), trastuzumab and lapatinib (*n* = 1), prior to pyrotinib treatment, and 17 (45.9%) patients had trastuzumab resistance. Pyrotinib treatment lines: 12 (32.4%) of the enrolled patients were on pyrotinib as a first-line treatment, 16 (43.2%) for second-line treatment, and nine (24.3%) for third-line treatment. Treatment: one patient (2.7%) was on pyrotinib alone, four patients (10.8%) were on pyrotinib + endocrine, and 32 patients (86.5%) were on pyrotinib + chemotherapy (12 with pyrotinib + capecitabine and 20 with pyrotinib + other chemotherapeutic agents). Single target therapy was used in 15 patients (40.5%) and double target therapy was used in 22 patients (59.5%). Initial dose of pyrotinib: seven patients (18.9%) started at ≤240 mg/d and 30 (81.1%) started at >240 mg/d. Nearly half of the patients (43.2%) were given pyrotinib at an initial standard dose of 400 mg/d. During the treatment period, five patients experienced dose reductions, and five patients were able to maintain the 320 mg/d or 400 mg/d dose without dose reductions or discontinuation after a gradual increase in dosage.

**Table 1:** Clinical baseline characteristics of 37 patients.

Characteristics	Patients, No (%) (*N* = 37)
Median age (range), years	50 (29–79)
<50	18 (48.6)
≥50	19 (51.4)
Menopause	
Yes	22 (59.5)
No	15 (40.5)
ECOG performance status	
0	8 (21.6)
1	26 (70.3)
2	3 (8.1)
Clinical stage	
III	2 (5.4)
IV	35 (94.6)
HR status	
Positive	16 (43.2)
Negative	21 (56.8)
Brain metastases	
Yes	6 (16.2)
No	31 (83.8)
Visceral metastasis	
Yes	31 (83.8)
No	6 (16.2)
Number of metastasis	37 (100)
≤2	25 (67.6)
>2	8 (21.6)
Previous anti-HER2 condition	
Yes	29 (78.4)
No	8 (21.6)
Previous use of lapatinib	
Yes	2 (5.4)
No	35 (94.6)
Trastuzumab resistance	
Yes	17 (45.9)
No	12 (32.4)
Pyrotinib treatment lines	
1	12 (32.4)
2	16 (43.2)
3	9 (24.3)
Treatment	
Pyrotinib + chemotherapy	32 (86.5)
Pyrotinib alone	1 (2.7)
Pyrotinib + endocrine	4 (10.8)
Combined with chemotherapy regimens	
Capecitabine	12 (32.4)
Other	20 (54.1)
Targeted therapies	
Single target	15 (40.5)
Double target	22 (59.5)
Initial dose of pyrotinib	
400 mg/d	16 (43.2)
320 mg/d	14 (37.8)
240 mg/d	5 (13.5)
160 mg/d	2 (5.4)
≤240 mg/d	7 (18.9)
>240 mg/d	30 (81.1)
Ki67	
≥15%	32 (86.5)
<15%	5 (13.5)

**Notes.**

ECOG, Eastern Cooperative Oncology Group; HR, hormone receptor; HER2, human epidermal growth factor receptor 2; mPFS, median progression-free survival; Single target, the treatment regimen contained only one targeted agent, pyrotinib; Double target, refers to regimen containing two targeted agents, pyrotinib and another targeted drug; Ki67, an immunohistochemical marker for assessing cell proliferation in malignant tumors and other proliferative conditions.

### Efficacy results

**Figure 2:**
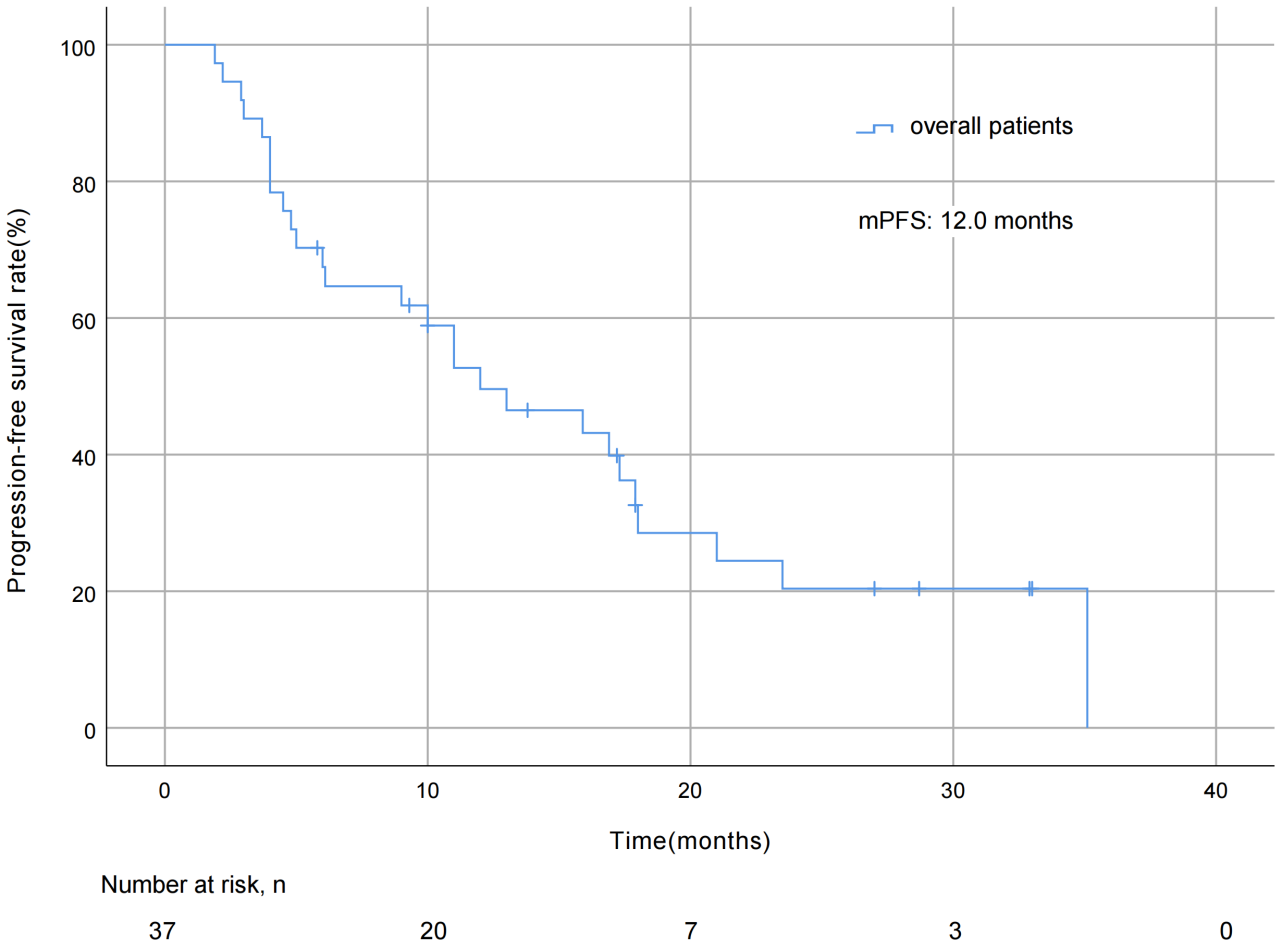
Progression free survival curve of all 37 patients.

As of October 7, 2022, the median duration of exposure to pyrotinib was 12.0 months, the median follow-up length was 29.7 months, with pyrotinib efficacy evaluated in all 37 patients and no patient deaths during the study period. The mPFS was 12.0 months (95% CI [5.8 ∼18.2] months; Fig. 2). As for best overall response (BOR) distribution, no patients achieved CR, 23 (62.2%) patients had PR, 12 (32.4%) patients had SD, and two (5.4%) patients experienced progression of disease (PD). The ORR was 62.2% and the DCR was 94.6% (Table 2).

Table 3 shows the PFS analysis by different subgroups. No significant differences were observed in mPFS (17.3 *vs* 10.0 months) between age groups (<50 *vs* ≥50 years) (*P* = 0.117), and no significant differences were observed between doses ≤240 mg/d and >240 mg/d (11.0 *vs* 13.0 months, *P* = 0.378). The differences in mPFS between menopausal and non-menopausal patients were also not significant (17.3 *vs* 11.0 months, *P* = 0.609). The mPFS of stage III and stage IV patients was similar (13.0 *vs* 11.0 months, *P* = 0.52). The mPFS of HR positive and HR negative patients were 9.0 and 13.0 months, respectively (*P* = 0.661), and the mPFS of the single target and double target groups were 15.9 months and 11.0 months, respectively (*P* = 0.644). There was no difference in mPFS between patients with or without visceral metastasis (11.0 months *vs* 13.0 months, *P* = 0.972). The mPFS of the Ki67 ≥ 15% group was 11.0 months, and the mPFS of the Ki67 < 15% group was not estimable (*P* = 0.184). Longer follow-up may be needed to accurately assess the trend of clinical difference between Ki67 expression level and survival benefit. The mPFS at ECOG score of 0–2 was 17.9 months, 9.0 months and 10.0 months, respectively, which may be due to differences in the physical conditions of the patient at the time of treatment, but this difference in mPFS by ECOG score was not statistically significant (*P* = 0.632). The difference in mPFS between patients with and without BMS was not statistically significant (5.0 months *vs* 15.9 months, *P* = 0.341). The mPFS of patients on pyrotinib alone and in combination with chemotherapy was 13.0 and 11.0 months, respectively, and not estimable for the group of patients on pyrotinib in combination with endocrine therapy, which had fewer patients who experienced disease progression. Numerically higher mPFS was seen in patients using pyrotinib in combination with capecitabine compared to the mPFS of patients using pyrotinib in combination with other chemotherapeutic agents (15.9 *vs* 6.1 months), but the difference was not statistically significant (*P* = 0.913).

**Figure 3:**
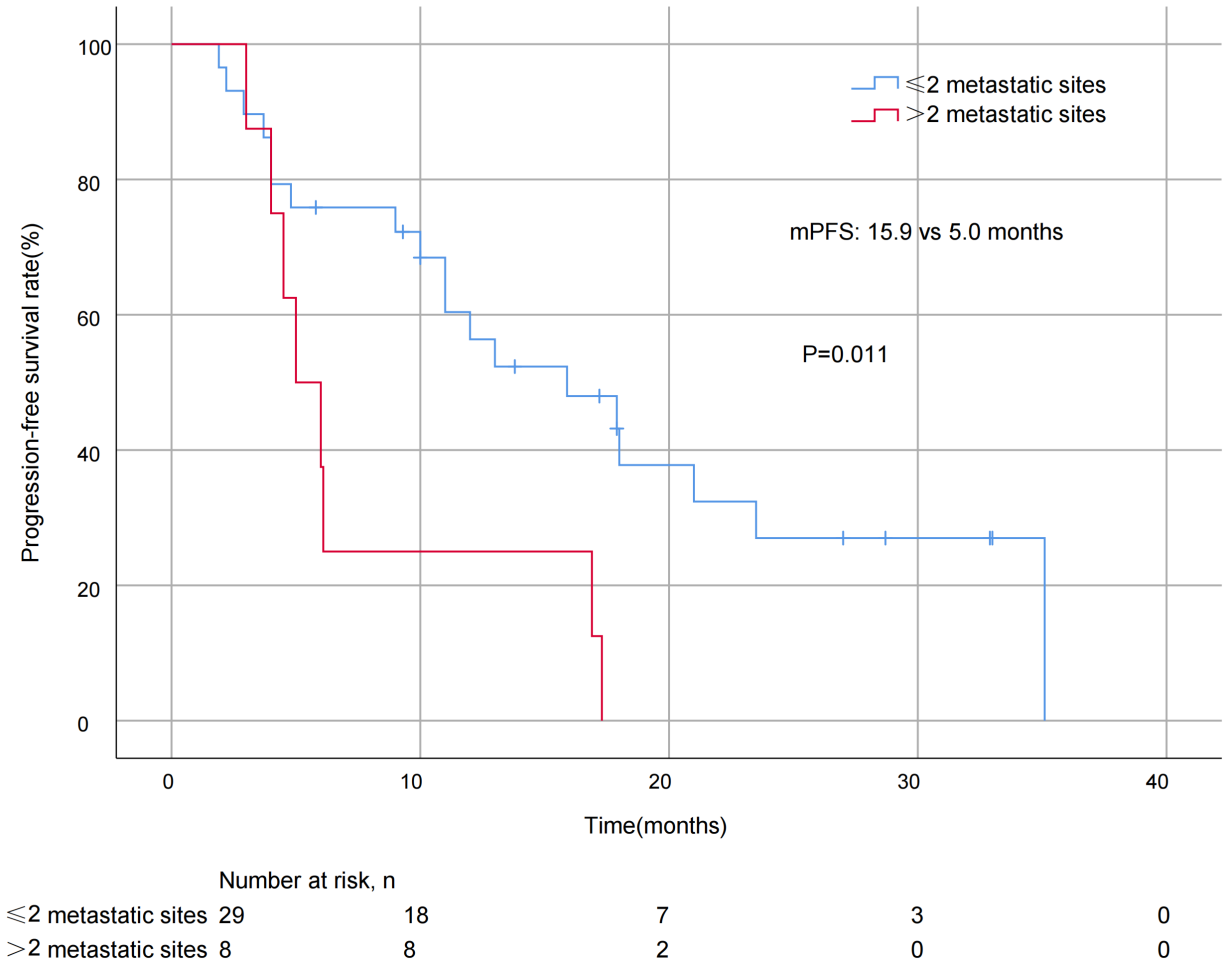
Progression free survival curves for patients with different numbers of metastatic sites.

**Figure 4:**
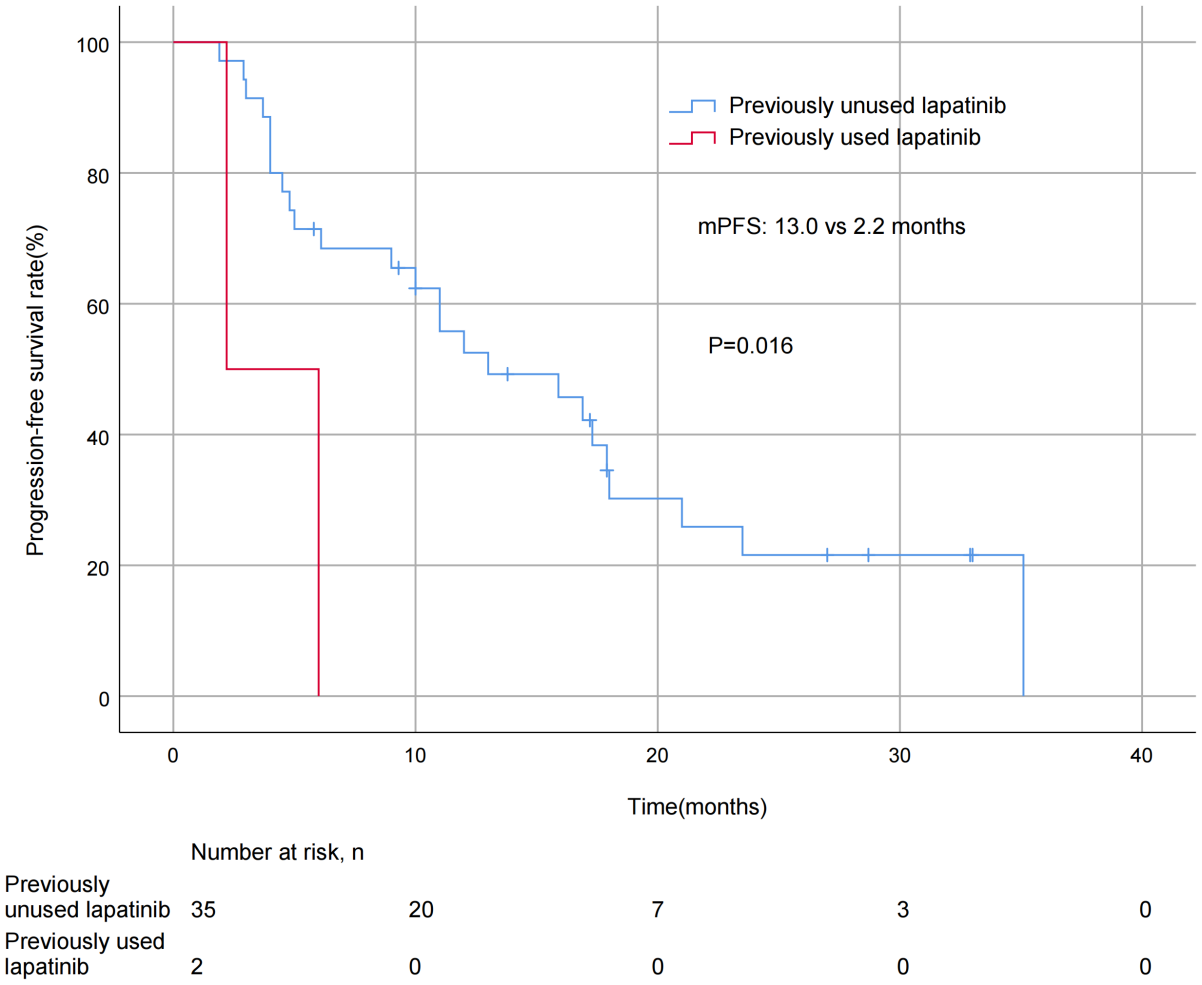
Progression free survival curves for patients with prior lapatinib use.

**Figure 5:**
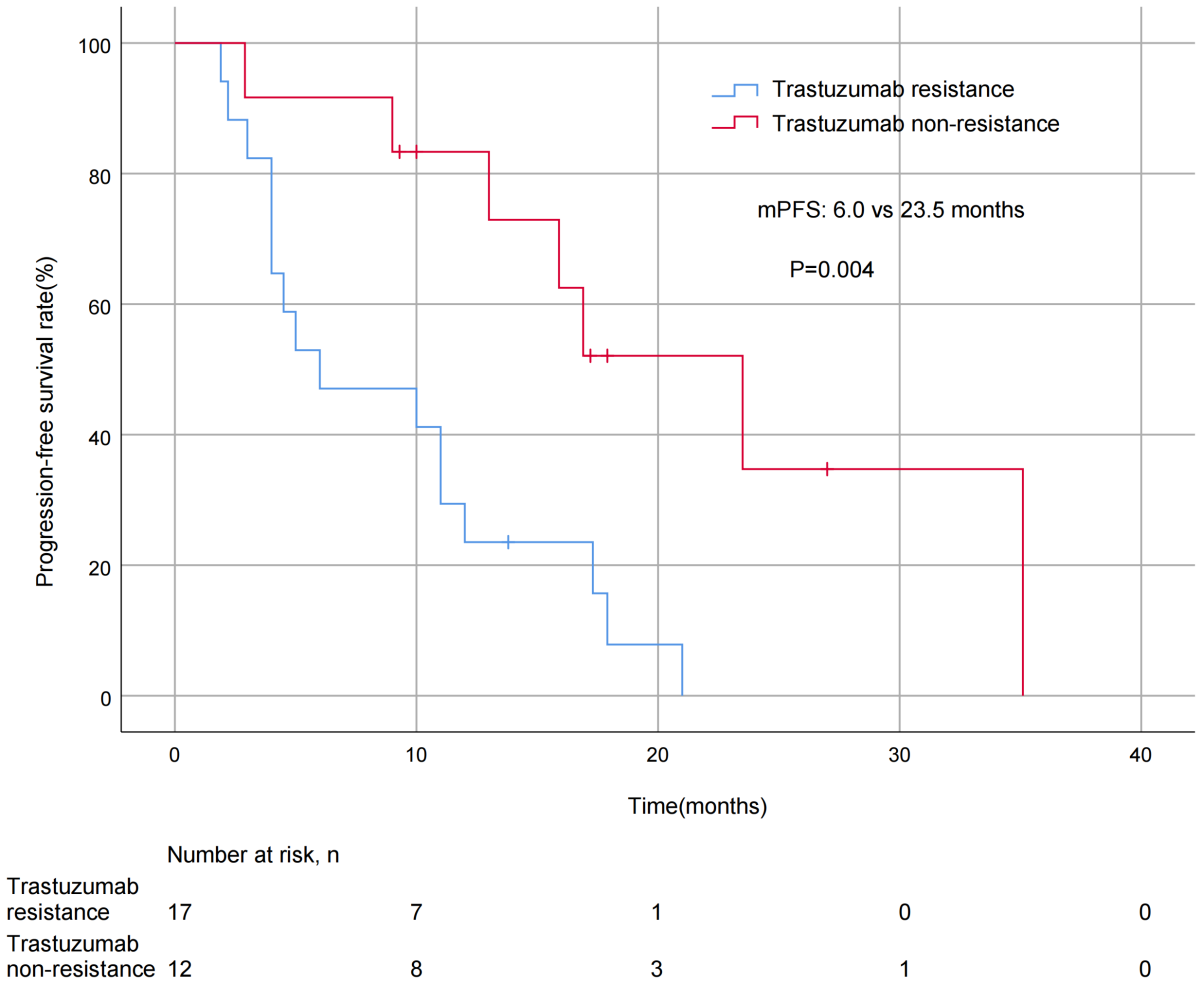
Progression free survival curves for patients with trastuzumab—resistance status.

**Figure 6:**
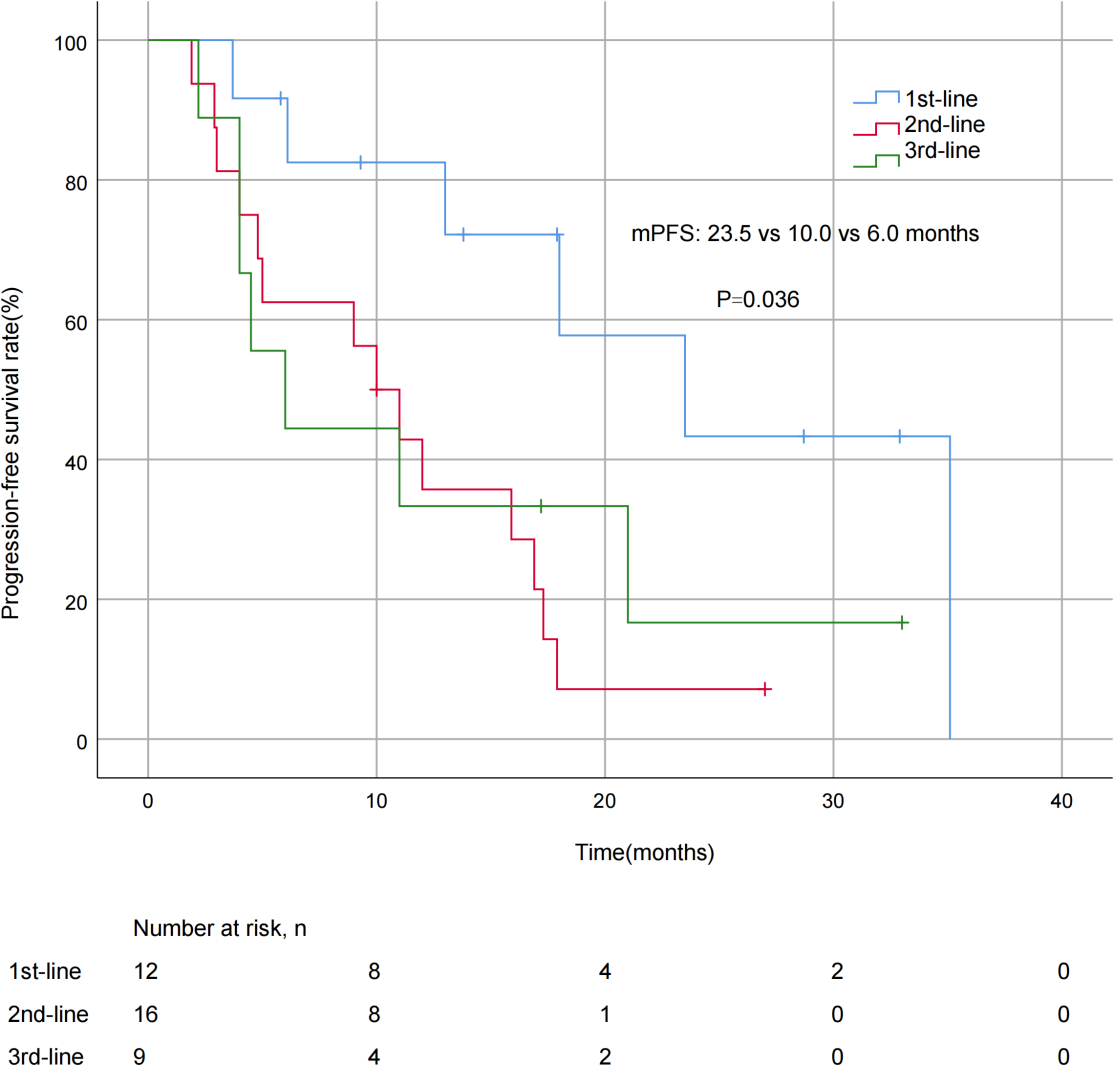
Progression free survival curves for patients with different treatment lines.

A significant difference in mPFS was found between patients with ≤2 metastatic sites (15.9 months) and > 2 metastases (5.0 months, *P* = 0.011; Fig. 3). The mPFS for patients who had previously received anti-HER2 medication was 18.0 months, and 12.0 months for patients who had not received anti-HER2 medication, but the difference was not statistically significant (*P* = 0.176). Patients who had not previously used lapatinib showed a different survival profile compared to those who had received lapatinib (13.0 *vs* 2.2 months, *P* = 0.016; Fig. 4). Furthermore, trastuzumab resistance status was a significant factor, with a clear difference in mPFS observed between trastuzumab intolerance and trastuzumab resistance patients (23.5 *vs* 6.0 months, *P* = 0.004; Fig. 5). A significant difference in mPFS was observed among the first-, second-, and third-line pyrotinib treatment groups(23.5 *vs* 10.0 *vs* 6.0 months, *P* = 0.036; Fig. 6).

**Table 2:** Overall efficacy of pyrotinib in the 37 patients.

Best response	Patients, No (%) (*N* = 37)
PR	23 (62.2)
SD	12 (32.4)
PD	2 (5.4)
ORR	23 (62.2)
DCR	35 (94.6)

**Notes.**

PR, partial response; SD, stable disease; PD, progressive disease; ORR, objective response rate; DCR, disease control rate.

**Table 3:** Between-group comparison of survival analysis of 37 patients.

Characteristic	mPFS(month)	*P*-value
Age		
<50	17.3	0.117
≥50	10.0	
Menopause		
Yes	17.3	0.609
No	11.0	
ECOG performance status		
0	17.9	0.632
1	9.0	
2	10.0	
Clinical stage		
III	13.0	0.52
IV	11.0	
HR status		
Positive	9.0	0.661
Negative	13.0	
Brain metastases		
Yes	5.0	0.341
No	15.9	
Visceral metastasis		
Yes	11.0	0.972
No	13.0	
Number of metastasis		
≤2	15.9	
>2	5.0	0.011
Previous anti-HER2 condition		
Yes	12.0	0.176
No	18.0	
Previous use of lapatinib		
Yes	2.2	0.016
No	13.0	
Trastuzumab resistance		
Yes	6.0	0.004
No	23.5	
Pyrotinib treatment lines		
1	23.5	0.036
2	10.0	
3	6.0	
Treatment		
Pyrotinib alone	13.0	0.26
Pyrotinib + chemotherapy	11.0	
Pyrotinib + endocrine	not estimable	
Combined with chemotherapy regimens		
Capecitabine	15.9	0.913
Other	6.1	
targeted therapies		
Single target	15.9	0.644
Double target	11.0	
Initial dose of pyrotinib		
≤240 mg/d	11.0	0.378
>240 mg/d	13.0	
Diarrhea degree		
Grade 1	15.9	0.291
Grade 2	13.0	
Grade 3	11.0	
Ki67		
≥15%	11.0	0.184
<15%	not estimable	

**Notes.**

ECOG, Eastern Cooperative Oncology Group; HR, hormone receptor; HER2, human epidermal growth factor receptor 2; mPFS, median progression-free survival; Single target, the treatment regimen contained only one targeted agent, pyrotinib; Double target, refers to regimen containing two targeted agents, pyrotinib and another targeted drug; Ki67, an immunohistochemical marker for assessing cell proliferation in malignant tumors and other proliferative conditions.

### Safety results

All 37 patients experienced different degrees of AEs during treatment with the pyrotinib-containing regimen. Diarrhea was the most common AE (97.3%), with 43.2% experiencing grade 1 diarrhea, 21.6% experiencing grade 2, and 32.4% experiencing grade 3. Other common AEs were hand-foot syndrome (51.4%) and anemia (54.1%), most of which were grade 1 and could be relieved by symptomatic treatment. Two patients experienced grade 1 hematochezia, and the symptoms disappeared after observation. Of the AEs experienced in this study, only diarrhea occurred as a grade 3 AE. There were no grade 4 AEs, and there were no AEs related to cardiac function (Table 4). Most AEs were mild and could be controlled by treating symptoms, indicating the pyrotinib-based regimen performs well in terms of safety. Based on this finding, the relationship between different maximum grades of diarrhea and mPFS was further analyzed, and no significant difference was observed (15.9 *vs* 13.0 *vs* 11.0 months, *P* = 0.291; Table 3).

## Discussion

BC is the most common type of malignant tumor in women, with 1/4 of BC patients having HER2 positivity. HER2 is a membrane receptor with tyrosine kinase activity, and the blockage of its signaling pathway is associated with tumor suppression (Vega Cano et al., 2022). Despite the widespread use of HER2-targeted therapies, patients with HER2-positive BC continue to face many challenges such as disease progression, treatment resistance, and brain metastasis. As a new, small-molecule anti-HER2 drug, pyrotinib crosses the cell membrane easier than macromolecule antibodies such as trastuzumab and irreversibly binds to intra-membrane targets, resulting in a stronger anti-HER2 effect (Qi et al., 2023). Several studies have shown that pyrotinib has strong efficacy in HER2-positive advanced BC (Miao et al., 2022; You et al., 2023; Li et al., 2023; Cao et al., 2023). Because pyrotinib has been added to the national reimbursement list, the cost-effectiveness of a pyrotinib regimen also gives the drug a significant advantage over other anti-HER-2 targeted drugs in China (Bao et al., 2022). We retrospectively reviewed the real-world data of pyrotinib treatment in HER2-positive stage III/IV BC patients in clinical practice, supplementing the results of clinical trials and providing a reference for further exploration of pyrotinib treatment patterns.

Pyrotinib was effective in controlling recurrent metastasis of HER2-positive metastatic BC, comparable to large real-world studies (Li et al., 2021; Liu et al., 2022). In the present study, the mPFS of patients with ≤2 metastases was significantly different from that of patients with >2 metastases (15.9 *vs* 5.0 months, *P* = 0.011). This may be related to fewer metastases equating to lower tumor invasiveness, which is consistent with the biological characteristics of HER2-positive breast cancer. The analysis of treatment line showed significant differences:the mPFS was 23.5 months for first-line treatment, compared to 10.0 months for second-line and 6.0 months for third-line treatment (*P* = 0.036). It is worth noting that in the present study, the mPFS of patients using pyrotinib as a first-line treatment appeared to be longer than those reported in previous studies (Zhang et al., 2021; Yin et al., 2022). These differences in efficacy in the first-line treatment setting suggest that early use of pyrotinib may better improve clinical outcomes in patients with advanced BC. However, even in the backline, pyrotinib still had strong anti-tumor efficacy, making it also an ideal treatment option for patients who have failed first-line therapy.

The application of trastuzumab significantly improved the prognosis of patients with HER 2 positive BC, but the problem of resistance was significant (De Melo Gagliato et al., 2016). In this study, 78.4% of patients had received anti-HER 2 medication, so we compared the effect of previous anti-HER 2 conditions on patients. Although the mPFS was not significantly different in untreated and pretreated patients (18.0 *vs* 12.0 months, *p* = 0.176), a finding which was similar to PHILA trial results (21.9 months; Ma et al., 2023b). The PHILA study included patients who had not received systemic antitumor therapy for recurrent or metastatic disease, and the patients in this study had progressed through multi-regimen therapy in the early stage. The overall prognosis of these patients was poor, but they still maintained a long survival, further reflecting the excellent antitumor activity of pyrotinib in patients with stage III/IV BC. We further explored the influence of trastuzumab resistance factors on the efficacy of pyrotinib. The mPFS in patients with trastuzumab intolerance was significantly different from that in those with trastuzumab resistance (23.5 *vs* 6.0 months, *P* = 0.004). It is worth noting that even in the presence of trastuzumab resistance, pyrotinib-based combination therapy could achieve not bad mPFS. This indicates pyrotinib may effectively overcome trastuzumab resistance, which may be because the HER2 small molecule inhibitor blocks the HER2 target region differently than trastuzumab and has a partially non-overlapping mechanism of action.

Results from previous clinical trials have shown that dual anti-HER2 regimens based on pyrotinib were expected to solve anti-HER2 resistance, with mPFS of 7.5–21.9 months (Xie et al., 2021; Xie et al., 2023; Ma et al., 2023b). The mPFS of patients on a dual-target regimen (pyrotinib combined with trastuzumab or inotuzumab) in the present study was 11.0 months, and high survival data were achieved in a wider clinical treatment context, which verified that pyrotinib combined with other targeted drugs could achieve synergistic effects by blocking HER2 signaling pathways. For patients who have failed to respond to trastuzumab, pyrotinib + capecitabine is first recommended. In this study, mPFS reached 15.9 months with pyrotinib + capecitabine, which was highly consistent with the results from the PHOEBE study (12.5 months) and the PHENIX study (11.1 months). Although there was no significant difference between pyrotinib + capecitabine and pyrotinib + other chemotherapy groups (*P* = 0.913), the clinical value of pyrotinib combined with other chemotherapy was still observed, providing a meaningful reference regimen for patients who are not tolerant to capecitabine.

This study exploratively analyzed the difference in efficacy of pyrotinib in lapatinib-treated and untreated BC patients. The mPFS was significantly different in the lapatinib untreated group compared to the treated group (13.0 *vs.* 2.2 months, *P* = 0.016), which may be related to pyrotinib’s ability to irreversibly inhibit HER 1, HER 2, and HER 4 simultaneously (Xuhong et al., 2019). However, the small sample size of the lapatinib-treated group (*n* = 2) may have amplified the magnitude of the difference, and the heavier treatment burden of these patients also resulted in a shorter mPFS. However, this group still achieved 2.2 months of mPFS after pyrotinib treatment, suggesting that pyrotinib may play a role in overcoming lapatinib resistance. Studies have shown that lapatinib pretreatment may induce HER2 mutations and weaken the efficacy of subsequent TKIs (Mao et al., 2025), which is consistent with the observed trend of reduced mPFS in this study. Lapatinib exposure history may affect the efficacy of pyrotinib, but large-scale studies are needed to confirm this.

Brain metastases (BMS) are considerably more likely to occur in patients with HER2-positive advanced BC, and with the widespread use of HER2-targeted therapy, the prognosis of these patients has been greatly improved (Zimmer, Swearingen & Anders, 2020). Compared with clinical trials, patients included in this study had more previous lines of treatment and a heavier visceral metastasis load (83.8%). The prognosis of such patients is usually poor. However, the mPFS of patients with brain metastasis on pyrotinib treatment regimens (5.0 months) was still consistent with the results of previously reported studies (Yan et al., 2022; Ma et al., 2023a), suggesting that pyrotinib showed better antitumor activity for refractory brain metastasis. Surprisingly, the mPFS of patients without BMS was 15.9 months, demonstrating the advantage of pyrotinib in inhibiting the development of BMS. Although a statistical difference was not reached (*P* = 0.341), these results suggest pyrotinib has potential clinical value in inhibiting BMS.

**Table 4:** Adverse events in preferred term by maximum grade in 37 patients.

AEs	All grades, *n* (%)	Grade 1, *n* (%)	Grade 2, *n* (%)	Grade 3, *n* (%)
Diarrhea	36 (97.3)	16 (43.2)	8 (21.6)	12 (32.4)
Nausea	12 (32.4)	7 (18.9)	5 (13.5)	0 (0.0)
Vomiting	1 (2.7)	1 (2.7)	0 (0.0)	0 (0.0)
Constipation	2 (5.4)	2 (5.4)	0 (0.0)	0 (0.0)
Abdominal pain	1 (2.7)	1 (2.7)	0 (0.0)	0 (0.0)
Fecal hidden blood	2 (5.4)	2 (5.4)	0 (0.0)	0 (0.0)
Hand-foot syndrome	19 (51.4)	13 (35.1)	6 (16.2)	0 (0.0)
White blood cell decline	12 (32.4)	8 (21.6)	4 (10.8)	0 (0.0)
Anemia	20 (54.1)	15 (40.5)	5 (13.5)	0 (0.0)
Neutrophil decline	9 (24.3)	3 (8.1)	6 (16.2)	0 (0.0)
AST increased	10 (27.0)	10 (27.0)	0 (0.0)	0 (0.0)
ALT increased	4 (10.8)	4 (10.8)	0 (0.0)	0 (0.0)

**Notes.**

AEs, Adverse Events; AST, glutamic oxaloacetic transaminase; ALT, glutamic oxaloacetic transaminase.

Diarrhea is a common adverse event associated with pyrotinib (Kioutchoukova & Lucke-Wold, 2023). Metabolomics studies have shown that intestinal microbiome imbalance and related metabolite changes are important factors in pyrotinib-induced diarrhea (Lai et al., 2023). The main adverse event associated with pyrotinib in this study was also diarrhea (97.3%), and no grade 4 AEs occurred. All diarrhea AEs in this study resolved, regardless of the degree, with symptomatic treatment or pyrotinib dose reduction, and these patients achieved a long mPFS. The mPFS of patients who experienced grade 3 AEs was comparable to the overall mPFS (11.0 *vs.* 12.0 months). Pyrotinib is well tolerated, safe and controllable, and improves patients’ quality of life. Notably, this study also found cases of fecal occult blood not previously reported, but it did not cause patient discomfort. The fecal occult blood may occur because pyrotinib inhibits EGFR targets associated with the gastrointestinal tract. This finding reminds clinicians to pay close attention to patients’ intestinal health during pyrotinib treatment in order to quickly detect and treat potential adverse reactions.

The results of this study provide reference information for clinicians in treatment planning and patient safety management and provide strong support for the clinical use of pyrotinib in the treatment of advanced BC. However, there are limitations of this study: small sample size, especially in some subgroups, such as the pyrotinib monotherapy and endocrine groups (five patients; 13.5%), BMS group (six patients; 16.2%), stage III group (two patients; 5.4%) and the group of patients who previously used lapatinib (two patients; 5.4%), affecting the statistical power; the retrospective design may introduce recall and selection bias; the follow-up period was relatively short, and overall survival was not observed; the heterogeneity of pyrotinib-based regimens represented a potential unmeasured confounder. These limitations mean the subgroup analysis results have limited generalizability and need to be referenced with caution. To definitively evaluate the efficacy of pyrotinib-based strategies and control for unmeasured confounders, future studies should consider randomized controlled trials that directly compare these regimens with the standard of care. Within the framework of such rigorous trials, exploring pyrotinib treatment strategies and validating predictive molecular biomarkers will be crucial for optimizing personalized therapy. The efficacy of pyrotinib combined with new targeted drugs is also a promising future research direction.

## Conclusion

Pyrotinib-based therapy has shown excellent performance in the treatment of HER2-positive stage III/IV BC patients and has a manageable safety profile.

##  Supplemental Information

10.7717/peerj.20524/supp-1 Supplemental Information 1 Raw data

## Additional Information and Declarations

### Data Availability

The following information was supplied regarding data availability: The raw data is available in the Supplemental Files.
